# High-salt intake affects retinal vascular tortuosity in healthy males: an exploratory randomized cross-over trial

**DOI:** 10.1038/s41598-020-79753-6

**Published:** 2021-01-12

**Authors:** Eliane F. E. Wenstedt, Lisanne Beugelink, Esmee M. Schrooten, Emma Rademaker, Nienke M. G. Rorije, Rosa D. Wouda, Reinier O. Schlingemann, Tien Y. Wong, Liffert Vogt

**Affiliations:** 1 Section of Nephrology, Department of Internal Medicine, Amsterdam Cardiovascular Sciences, A3-272, Amsterdam UMC, University of Amsterdam, location AMC, Meibergdreef 9, Amsterdam, 1105 AZ The Netherlands; 2 Department of Ophthalmology, Amsterdam UMC, University of Amsterdam, Meibergdreef 9, Amsterdam, 1105 AZ The Netherlands; 3 Department of Ophthalmology, University of Lausanne, Jules-Gonin Eye Hospital, Fondation Asile des Aveugles, Avenue de France 15, CP 5143, 1002 Lausanne, Switzerland; 4 Singapore Eye Research Institute, Singapore National Eye Centre, 20 College Road Discovery Tower, Level 6, The Academia, Singapore, 169856 Singapore

**Keywords:** Predictive markers, Nutrition, Hypertension

## Abstract

The retinal microcirculation is increasingly receiving credit as a relatively easily accessible microcirculatory bed that correlates closely with clinical cardiovascular outcomes. The effect of high salt (NaCl) intake on the retinal microcirculation is currently unknown. Therefore, we performed an exploratory randomized cross-over dietary intervention study in 18 healthy males. All subjects adhered to a two-week high-salt diet and low-salt diet, in randomized order, after which fundus photographs were taken and assessed using a semi-automated computer-assisted program (SIVA, version 4.0). Outcome parameters involved retinal venular and arteriolar tortuosity, vessel diameter, branching angle and fractal dimension. At baseline, participants had a mean (SD) age of 29.8 (4.4) years and blood pressure of 117 (9)/73 (5) mmHg. Overall, high-salt diet significantly increased venular tortuosity (12.2%, p = 0.001). Other retinal parameters were not significantly different between diets. Changes in arteriolar tortuosity correlated with changes in ambulatory systolic blood pressure (r = − 0.513; p = 0.04). In conclusion, high-salt diet increases retinal venular tortuosity, and salt-induced increases in ambulatory systolic blood pressure associate with decreases in retinal arteriolar tortuosity. Besides potential eye-specific consequences, both phenomena have previously been associated with hypertension and other cardiovascular risk factors, underlining the deleterious microcirculatory effects of high salt intake.

## Introduction

Salt intake in the Western world generally exceeds the amount as recommended by guidelines^1^. The effect that this may have on microcirculatory vessels is important to establish, both with regard to the link to hypertension development as well as to expose potential blood pressure-independent deleterious effects. In this regard, the microcirculation of the retina is of interest, due to its relatively easy accessibility as well as because of its proven value for cardiovascular risk assessment^2^.

Evidence for an effect of salt on the retinal microcirculation remains scarce. In rats, a low-salt diet reduces vascular endothelial growth factor and neovascularization in the retina^3^. Evidence in humans consists of only one cross-sectional study that shows a relation between urine sodium excretion and retinal vessel wall thickness and cross-sectional area in hypertensive patients^4^. For other microcirculatory vascular beds, like those of the skin, muscle, and sublingual space, the effect of salt has been shown to involve for example rarefaction (reduced blood vessel density) and reduced arteriolar vessel diameter^5–10^. The effect of a dietary salt intervention on the microcirculation of the retina, however, remains unknown.

Especially given the fact that the retinal vascular bed is considered to be a good reflection of vascular beds of other organs^2,11–13^, specifically in the context of hypertension and cardiovascular disease^2,14–16^, the effect of salt on the retinal microcirculation deserves exploration. In the Singapore Malay Eye study, a population-based cross-sectional study of 3280 participants, increased venular tortuosity (i.e. “twisted blood vessels”) and decreased arteriolar tortuosity were significantly associated with hypertension^17^. This is supported by other studies that showed a relation between hypertension and retinal vessel tortuosity^18–23^. Also other types of retinal microcirculatory changes have been associated with hypertension, among which reduced fractal dimension and reduced arteriolar branching angle^24–29^.

We aimed to study the effect of experimental high salt intake on the retinal microcirculation, involving venular and arterial tortuosity and other retinal vascular parameters including fractal dimension, arteriolar diameter, length diameter ratio and branching angle.

## Results

**Figure 1:**
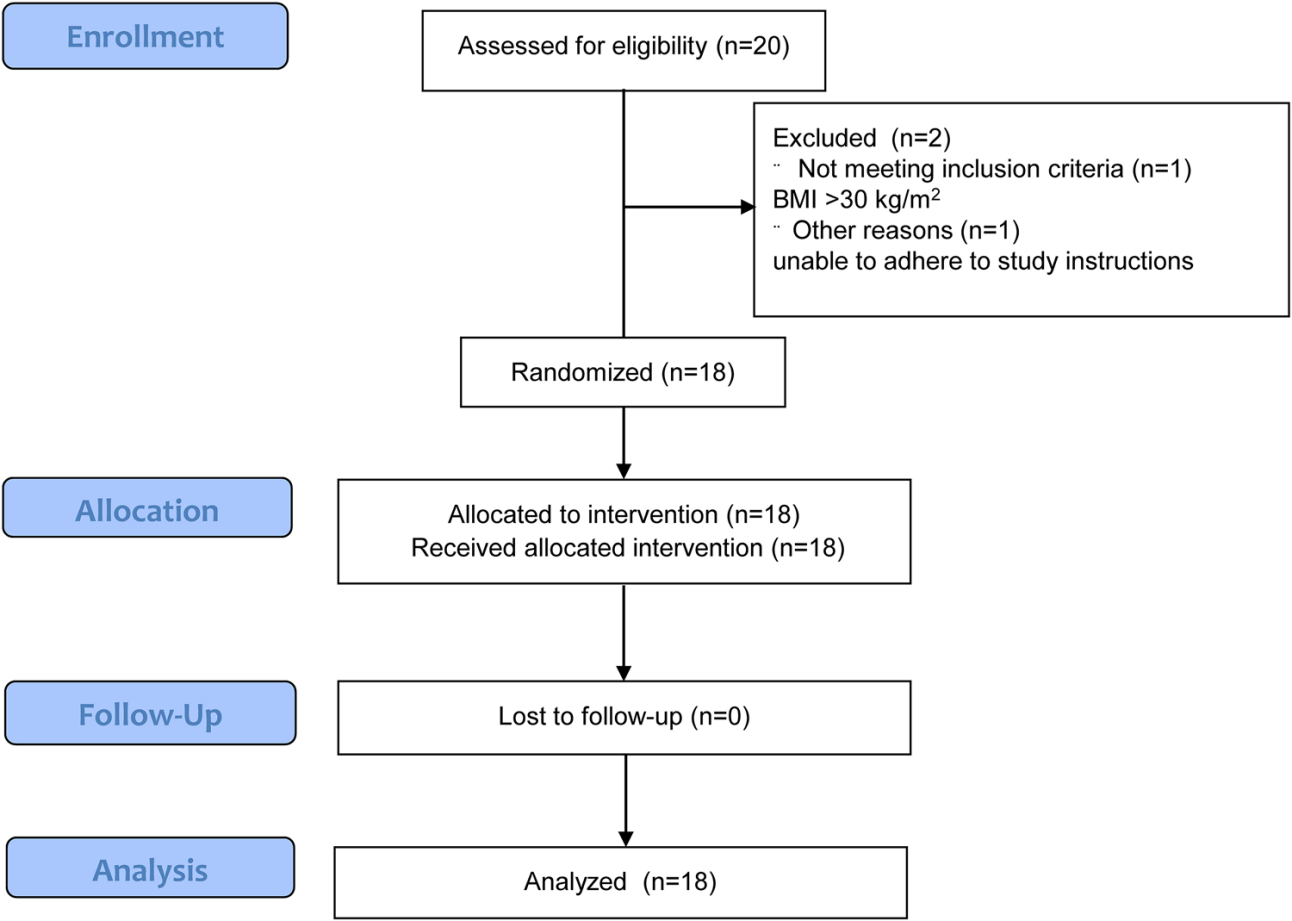
Flowchart of inclusion of participants.

Between October 2016 and April 2017, we screened 20 healthy male subjects (Fig. 1). One subject failed to meet the inclusion criteria, and one subject was excluded due to failure to adhere to the study protocol, therefore 18 subjects remained for randomization and analysis, 10 of which were randomized to start with a high-salt diet and 8 to start with a low-salt diet. Sixteen subjects were of European descent, two of Arabic descent. Mean (SD) age at baseline was 29.8 (4.4) years, mean blood pressure at baseline was 118 (9)/73 (5) mmHg. Mean salt intake as estimated from 24-h urine collections equaled ± 9 g of salt/day (150 (91) mmol sodium). Table 1 shows the baseline characteristics in detail (measured at a screening visit before commencement of the diets).

**Table 1:** Baseline characteristics at screening visit (before commencement of the diets).

**Healthy male subjects N = 18**	
Age (years)	29.8 (4.4)
Weight (kg)	80.6 (8.5)
BMI (kg/m^2^)	24.4 (2.6)
Waist-to-hip ratio	0.92 (0.05)^a^
**Plasma**	
Sodium (mmol/l)	140 (1)
Potassium (mmol/l)	4.1 (0.02)
Osmolality (mOsm/kg)	293 (2)
eGFR (ml/min per 1.73m^2^)	106 (12)
**24 h urine**	
Sodium (mmol/24 h)	150 (91)^a^
Potassium (mmol/24 h)	83 (54)^a^
Sodium/potassium ratio	1.9 (0.7)
Creatinine (mmol/24 h)	17.0 (4.6)
Creatinine clearance (ml/min)	141 (42)
**Office BP**	
SBP (mmHg)	118 (9)
DBP (mmHg)	73 (5)
MAP (mmHg)	88 (6)
Heart rate (bpm)	64 (9)

All values are expressed as mean (SD), unless marked otherwise.

*BP* blood pressure, *SBP* systolic blood pressure, *DBP* diastolic blood pressure, *MAP* mean arterial pressure.

^a^Values are expressed as median (interquartile range).

The outcome measurements after low-salt diet and high-salt diet are shown in Table 2. Mean salt intake during low-salt diet equaled ± 2 g of salt/day (32.8 mmol sodium) and during high-salt diet equaled ± 17 g of salt/day (286.3 mmol sodium) (p < 0.001). There was no significant difference in systolic blood pressure (121 (7) vs. 119 (9) mmHg; p = 0.13) or diastolic blood pressure (70 (5) vs. 70 (4) mmHg; p = 0.89) between high-salt diet and low-salt diet. Weight significantly increased after high-salt diet with a mean of 1.8 kg or 2.3% (p < 0.001).

**Table 2:** Outcome measurements after low and high salt diet.

	Low salt diet N = 18	High salt diet N = 18	P value
Weight	79.1 (8.4)	80.8 (8.1)	< 0.0001
**Plasma**			
Sodium (mmol/l)	140 (2)	141 (1)	0.13
**24 h urine**			
Volume (ml/24 h)^a^	1948 (1421)	2446 (1307)	0.18
Sodium (mmol/24 h)^a^	32.8 (27.5)	286.3 (189.4)	0.0002
Potassium (mmol/24 h)^a^	90.5 (47.7)	113.8 (36.4)	0.018
Sodium/potassium ratio	0.4 (0.2)	2.7 (0.8)	< 0.0001
**Ambulatory BP measurement**^**b**^	(n = 16)^b^	(n = 16)^b^	
SBP (mmHg)	119 (9)	121 (7)	0.13
DBP (mmHg)	70 (4)	70 (5)	0.89
MAP (mmHg)	93 (4)	94 (4)	0.28
Heart rate (bpm)	62 (7)	64 (5)	0.15
**Retinal outcomes**^**a**^			
CRAE-B	159.54 (31.28)	160.99 (44.12)	0.95
CRAE	166.29 (35.50)	161.55 (37.27)	0.53
CRVE-B	228.38 (48.06)	223.45 (57.22)	0.33
CRVE	238.23 (45.30)	236.84 (46.84)	0.33
AVR-B	0.69 (0.06)	0.70 (0.09)	0.53
AVR	0.69 (0.06)	0.70 (0.08)	0.31
Fractal dimension	1.45 (0.05)	1.44 (0.07)	0.38
Arteriolar tortuosity × 10^5^	5.44 (1.82)	5.55 (2.03)	0.62
Venular tortuosity × 10^5^	5.49 (0.72)	6.16 (0.79)	0.001*
Arteriolar branching angle	89.10 (16.06)	85.61 (17.14)	0.35
Venular branching angle	77.09 (19.50)	77.14 (13.76)	0.59
LDRa	13.48 (10.15)	15.21 (10.63)	0.079
LDRv	10.99 (9.46)	10.52 (5.44)	0.112

All values are expressed as mean (SD), unless marked otherwise.

*BP* blood pressure, *SBP* systolic blood pressure, *DBP* diastolic blood pressure, *MAP* mean arterial pressure, *CRAE-B* central retinal equivalent in zone B, *CRAE* central retinal equivalent in zone C, *CRVE(-B)* central retinal equivalent in zone B or C, *AVR(-B)* arteriovenous ratio in zone B or C, *LDRa* length diameter ratio arteriole.

^a^Values are expressed as median (interquartile range).

^b^Two participants had no ambulatory blood pressure measurements due to device malfunction.

**Figure 2:**
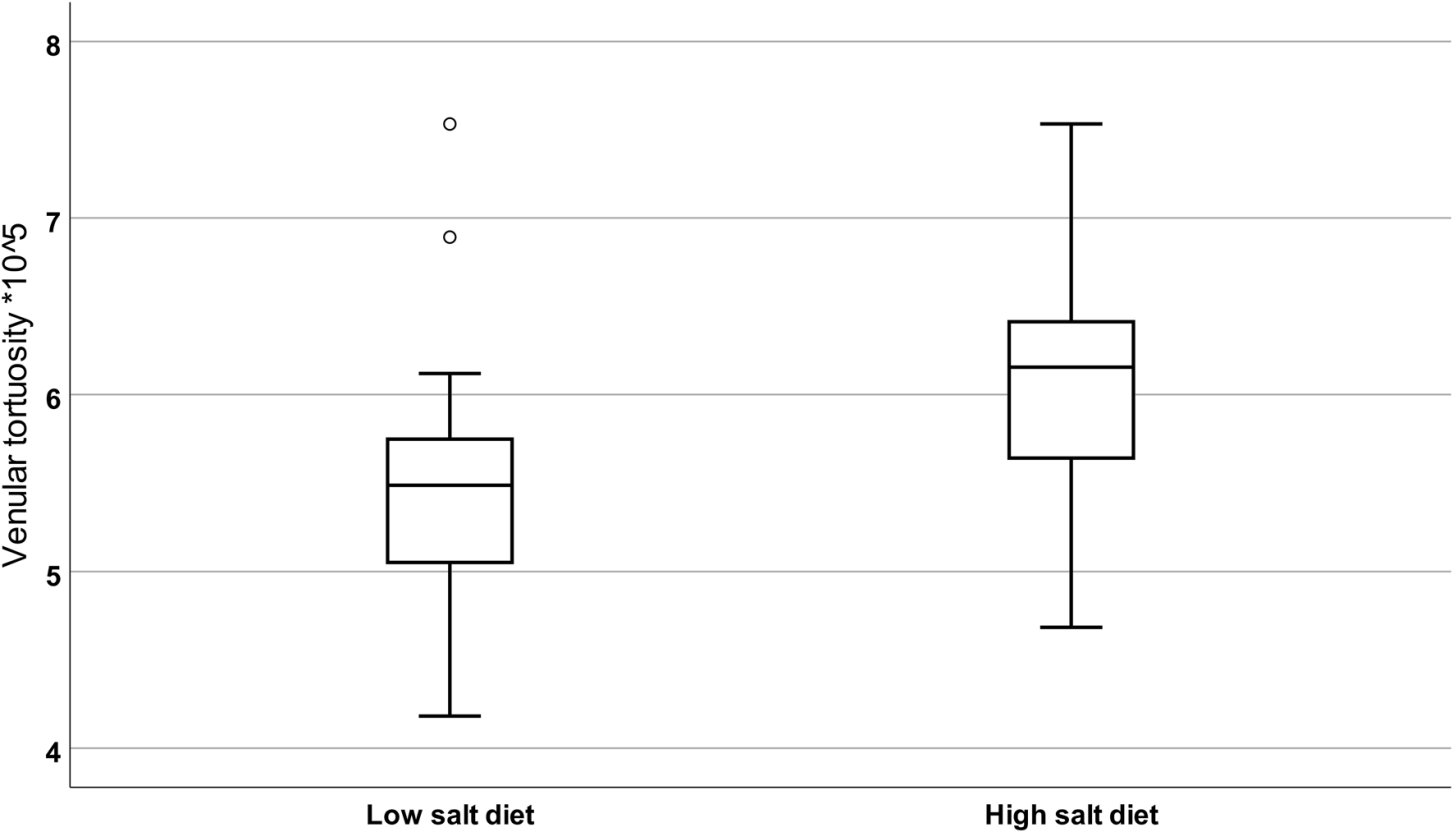
Boxplot of venular tortuosity after low salt diet and high salt diet. Venular tortuosity was significantly higher after the high salt diet (P = 0.001).

**Figure 3:**
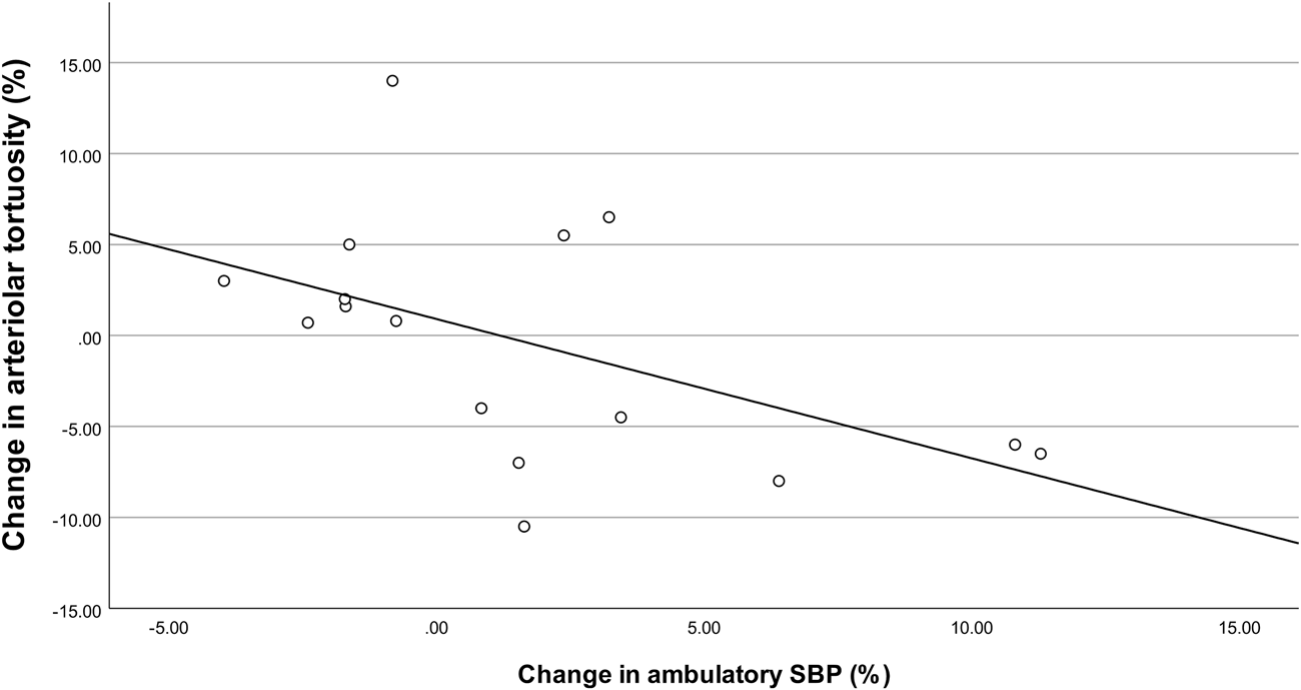
Scatterplot of the correlation between change in arteriolar tortuosity and change in ambulatory SBP. Changes were calculated as the values of (high-salt diet − low-salt diet)/(low-salt diet)* 100. There was a significant correlation between arteriolar tortuosity and the ambulatory SBP (r = − 0.513; p = 0.042). SBP, systolic blood pressure.

In the retina, high-salt diet significantly increased venular tortuosity with a difference of 0.67 or 12% (p = 0.001 (p < 0.0038 with Bonferroni correction); Fig. 2). This effect appeared to be independent of blood pressure, since a significant effect of diet remained after adding ambulatory systolic blood pressure changes to the model (p = 0.001). There were no significant differences in the other retinal vascular parameters between diets. There were also no correlations between retinal vascular parameters and 24-h urine sodium or potassium excretion. Overall, there were no period effects or carry-over effects. There was a significant correlation between arteriolar tortuosity and ambulatory systolic blood pressure, (r = − 0.513; p = 0.042, Fig. 3). Exploratory subgroup analysis revealed that arteriolar tortuosity decreased in subjects with an increased ambulatory SBP with -10.76 (17.92)%, while arteriolar tortuosity in subjects without an increase in ambulatory SBP did not change (Supplementary Table 1, Supplementary Fig. 1). Other retinal vascular parameters did not differ between these subgroups.

## Discussion

The aim of this study was to assess the influence of experimental high and low dietary salt intake on the retinal microcirculation in healthy male subjects. We demonstrated that a high-salt diet significantly increases retinal venular tortuosity, and that systolic blood pressure increases correlate with decreases in retinal arteriolar tortuosity.

The retinal microcirculation is considered to reflect microcirculatory vessels of other organ systems and is, amongst others, a well-suited marker for cardiovascular risk prediction^2,16^. Specifically, the observed salt-induced changes in the present study—increased venular tortuosity and decreased arteriolar tortuosity—have been shown to be present in individuals with hypertension in the Singapore Malay Eye Study^17,26^. Also in another cross-sectional study of 218 subjects, decreased retinal arteriolar tortuosity correlated with higher blood pressure^20^. Furthermore, in the Australian Heart Eye Study (AHES), an observational study involving 1680 participants, decreased retinal arteriolar tortuosity was associated with greater odds of coronary artery disease in men^22^. The Beaver Dam Eye Study, a case–control study with 4926 participants, showed similar results and demonstrated that decreased retinal arteriolar tortuosity was significantly associated with ischemic heart disease ischemic heart disease-related death independently from other cardiovascular risk factors such as diabetes, cholesterol levels, BMI or smoking^26^. A recent meta-analysis showed that genetic determinants of retinal arteriolar tortuosity are also linked to cardiovascular health^30^. We performed exploratory correlation analyses on the relation between the retinal changes and previously published sublingual changes in this population^10^, however no associations were found (data not shown), likely due to differences inherent to the specific method (e.g. measurement of sublingual microvascular vessels with SDF vs. intermediate vessels in the retina, and specific distinct microcirculatory parameters with each method).

The underlying mechanisms of our observations in changed tortuosity remain uncertain. Changes in vascular tortuosity may be the result of either an increase in vessel length due to increased intravascular volume, the result of vascular remodeling, or both. In this study, vascular tortuosity was calculated as “the integral of the curvature square along the path of the vessel, normalized by the total path length”^31^ and was therefore already corrected for vessel length. Furthermore, if the increased tortuosity was the result of an increased intravascular volume, we would also expect an increase in vascular caliber, which was not present in our study. Also the authors of the Singapore Malay Study hypothesized that changes in retinal vascular tortuosity may be the result of another type of structural vascular remodeling than changes in vessel caliber, given the independent association between changes in tortuosity and blood pressure in their study^17^. In this regard, it may be noted that it is established that high salt exposure has direct adverse effects on the vascular endothelium as well as on the endothelial surface layer (glycocalyx), potentially underlying vascular remodeling^32^.

Besides reflecting potential systemic microcirculatory changes, the high-salt-induced increase in retinal venular tortuosity may also have eye-specific consequences. Although the follow-up of our study was too short to detect such possible deleterious consequences in the retina, amongst others, increased retinal venular tortuosity is found in diabetic retinopathy^12,33^, and correlates with reduced retinal vasodilation^34^. Additionally, increased venular tortuosity is associated with an increased concentration of vascular endothelial growth factor A (VEGF-A) in the eye^13^. Interestingly, both VEGF-A as well as VEGF-C (another subtype of VEGF), have been shown to increase in response to high salt^35–38^. This may have important consequences for the eye, given the important roles of VEGF-A in retinal diseases, and the wide-spread therapeutic use of VEGF-A inhibitors^39^.

To our knowledge, we are the first to conduct a dietary intervention study to assess the short term influence of salt on the retinal microcirculation. We used a well-controlled randomized experimental design with a dietary protocol that adheres to recent guidelines^40^. Retinal microcirculatory changes were assessed by experienced and blinded researchers using a semi-automated computer-assisted program (SIVA, version 4.0). Despite these strengths, certain limitations need to be addressed. First, the sample size was relatively small and retinal endpoints were the secondary exploratory outcomes of a previously published trial on sublingual microcirculation^10^. More subtle effects may be missed in small study populations, and data that show trends without statistical significance may have encountered a power problem (e.g. length to diameter ratio in the present study). Second, because the studied subjects were all healthy males, the observed results may not be applicable to other groups, such as women, patients with cardiovascular disease or patients with diabetes. Also, the increase in salt intake was accompanied by an unintended increase in potassium intake. Dietary potassium has been linked to hypertension, and increased potassium intake may lower blood pressure^41^. However, the increase in salt intake (5.5 g sodium) largely exceeded the increase in potassium intake (0.6 g potassium), also shown by a significant increase of the sodium/potassium ratio. Furthermore, no correlation was found between 24-h potassium excretion and retinal measurements. It is therefore improbable that the changes we found in this study are the direct effect of an increased potassium intake. Finally, the retinal circulation differs from other vascular beds, since it has a blood-retinal barrier similar to the brain microvasculature, and does not have autonomic innervation, but is regulated by local auto regulatory mechanisms only^42–44^. Although retinal microvascular arteriolar and venular diameter may be relevant and sensitive markers for microvascular (dys)function^45^, generalization of other retinal microvascular parameters—in this case tortuosity measurements—to other vascular beds should be done with caution.

In conclusion, we show that a 2-week high-salt diet affects retinal vascular tortuosity in healthy males. Venular tortuosity is increased, and decreases in arteriolar tortuosity associate with increases in systolic blood pressure. Both phenomena have been associated with deleterious outcomes such as hypertension and other cardiovascular risk factors by others, underlining the deleterious effects of salt intake on the microcirculation, and potential eye-specific consequences need further consideration.

## Methods

### Participants and ethics approval

In this prospective, randomized experimental interventional cross-over study, we included healthy, nonsmoking males between the age of 18 and 40 years. Exclusion criteria were hypertension (untreated office blood pressure ≥ 140/90 mmHg), obesity (body mass index ≥ 30 kg/m^2^), a history of renal or auto-immune disease, eye surgery, glaucoma or retinal disorders, major illness in the last three months, malignancy in the last five years (with the exception of basal cell carcinoma) and substance abuse. The study was performed at the Amsterdam University Medical Center location Academic Medical Center, The Netherlands between October 2016 and April 2017 according to the principles of the Declaration of Helsinki^46^. All subjects were recruited by local advertisement and provided a written informed consent. The study was approved by local ethics commission (METC AMC) and was registered at the Netherlands Trial Register (NTR4785). This report was written in accordance with the CONSORT guidelines^47^.

### Study design

All subjects were enrolled by the research physicians. After the screening visit and informed consent, subjects received, in a randomized order, both a high-salt diet (> 12 g NaCl/day) and a low-salt diet (< 3 g NaCl/day), each for fourteen consecutive days each, without a washout period. Subjects were provided with a diet list. To keep intake of other nutrients (e.g., potassium, protein, carbohydrates and fats) stable, subjects were asked to replace the food products of their habitual diets with a high salt content for the same product with low salt content, or vice versa. Randomization was performed via sealed opaque envelopes in blocks of four. Dietary compliance was verified with 24-h urine collection during the diets at day seven and day eleven of each diet. On day fifteen of both diets, subjects visited our research facility after an overnight fast for fundus photography, hemodynamic measurements, blood samples and a 24-h urine collection. Diet status was not masked for the study subjects or observers during follow-up. However, blinding was ensured for data extraction from the images of the retinal microcirculation.

### Retinal microcirculatory measurements

The retinal microcirculatory properties were assessed via fundus photography of the right eye with a TRC-50DX fundus camera (Topcon Europe Medical B.V., Capelle aan de IJssel, The Netherlands) based on the principle of monocular indirect ophthalmoscopy, performed by a professional fundus photographer at our research facility. To improve the quality of the photographs, subjects received one drop of tropicamide for pupil dilation. All photographs were graded via Singapore I Vessel Assessment (SIVA version 4.0; National University of Singapore, Singapore)^48^ at the Singapore National Eye Centre Ocular Reading Centre and provided the following parameters: arteriolar and venular tortuosity, central retinal artery equivalent (as a measure for arteriolar diameter), central retinal vein equivalent (as a measure for venular diameter), arteriovenous ratio, fractal dimension, arteriolar and venular branching angle and arteriolar and venular length diameter ratio. Arteriolar and venular diameter and arteriovenous ratio were measured in the region between 0.5 and 1 optic disk diameter from the optic disk margin (zone B), as well as the region between 1 and 2 optic disk diameter from the optic disk margin (zone C)^31^. Researchers were blinded for diet allocation.

### Hemodynamic measurements

Hemodynamic parameters were measured at baseline and on day fifteen of each diet. At day fourteen of each diet, 24-h ambulatory measurements of systolic and diastolic blood pressure, mean arterial pressure and heart rate were recorded at the non-dominant arm (Mobil-O-Graph 24 h PWA Monitor; I.E.M. GmbH, Mannheim, Germany). These parameters were measured with a 15-min interval during day time and a 30-min interval during night time.

### Laboratory testing

Blood samples were taken at baseline and on day fifteen for laboratory testing, including sodium, potassium, creatinine, glucose and osmolality. Twenty four-hour urine sample testing included sodium, potassium and creatinine. Both blood and urine samples were analyzed with the COBAS C8000 Modular Analyzer (Roche Diagnostics GmbH, Mannheim, Germany).

### Sample size calculation

The primary aim of this study was to assess capillary recruitment from capillary density measurements with sublingual Sidestream Dark Field-imaging, the results of which were recently published^10^. The predefined secondary aim was to assess the effect of dietary salt intake on the retinal microcirculation. Sample size was calculated based on the primary endpoint of capillary recruitment based on results of a previously conducted clinical experiment. Given a standard deviation (SD) of 12.0, at least 18 subjects per group were needed to demonstrate an effect of 8.4% (2-sided t-test, power 80%, alpha-error 5%).

### Statistical analysis

Data were analyzed with IBM SPSS Statistics (version 25.0, IBM, USA). Continuous variables were expressed as mean and SD if normally distributed, or median and interquartile range if data were not normally distributed. Paired samples t-test or Wilcoxon signed rank test were used to compare outcomes of both diets. Bonferroni correction was applied to correct for multiple testing of retinal parameters. Spearman correlation was used to test for correlation between retinal parameters and changes in sodium excretion, body weight and blood pressure. A linear mixed model with diet as repeated factor and ambulatory systolic blood pressure as covariate was performed to assess whether potential differences between diets depended on blood pressure. We checked for period and carry-over effects^49^. An exploratory subgroup analysis was performed, dividing between subjects with vs. without an increase in ambulatory systolic blood pressure after high-salt diet. A two-tailed P-value of less than 0.05 was considered statistically significant.

## Supplementary Information

Supplementary Information

## Data Availability

The datasets generated during and/or analysed during the current study are available from the corresponding author on reasonable request.
