# Supplementary material for: High-salt intake affects retinal vascular tortuosity in healthy males: an exploratory randomized cross-over trial
Source: Sci Rep. 2021 Jan 12;11:801. doi: 10.1038/s41598-020-79753-6 (PMC7803999; doi:10.1038/s41598-020-79753-6)

SUPPLEMENTARY MATERIAL

HIGH-SALT INTAKE AFFECTS RETINAL VASCULAR TORTUOSITY IN HEALTHY MALES – AN EXPLORATORY RANDOMIZED CROSS-OVER TRIAL

Eliane F.E. WENSTEDT[1,*], Lisanne BEUGELINK[1,*], Esmee M. SCHROOTEN[1], Emma RADEMAKER[1], Nienke M.G. RORIJE[1], Rosa D. WOUDA[1], Reinier O. SCHLINGEMANN[2,3], Tien Y. WONG[4], Liffert VOGT[1]

[1]Amsterdam UMC, University of Amsterdam, Department of Internal Medicine, Section of Nephrology, Amsterdam Cardiovascular Sciences, Meibergdreef 9, Amsterdam 1105AZ, The Netherlands
[2]Amsterdam UMC, University of Amsterdam, Department of Ophthalmology, Meibergdreef 9, Amsterdam 1105AZ, The Netherlands
[3]Jules-Gonin Eye Hospital, Fondation Asile des Aveugles, Department of Ophthalmology, University of Lausanne, Avenue de France 15, CP 5143, CH – 1002 Lausanne, Switzerland
[4]Singapore Eye Research Institute, Singapore National Eye Centre, 20 College Road Discovery Tower, Level 6 The Academia, Singapore 169856, Singapore

***: equal contribution**

Supplementary Table 1. Outcome measurements after median split delta ambulatory SBP

	No increase SBP N=8	Increase SBP N=8	P value
ΔWeight (%)	2.0 (1.3)	3.0 (1.0)	0.14
Δ24 h urine (%)			
Volume[a]	30.56 (69.44)	6.03 (189.13)	0.60
Sodium[a]	794.07 (788.54)	912.48 (1305.56)	0.40
Potassium[a]	18.29 (61.33)	31.11 (62.69)	0.46
Sodium/potassium ratio	718.14 (419.61)	780.25 (420.58)	0.77
ΔAmbulatory BP measurement (%) [b]			
SBP	-1.45 (1.89)	5.14 (3.91)	0.001*
DBP	-0.12 (3.07)	0.69 (6.68)	0.76
MAP	-0.80 (1.91)	3.21 (4.98)	0.06
HR	6.02 (9.70)	2.18 (9.98)	0.44
ΔRetinal outcomes (%) [a]			
CRAE-B	-5.6165 (24.17)	1.5750 (10.40)	0.18
CRAE	-4.8867 (18.72)	0.5380 (5.38)	0.33
CRVE-B	-4.7846 (17.76)	-0.2576 (5.83)	0.42
CRVE	-3.9002 (23.50)	-1.0523 (7.33)	0.32
AVR-B	-0.0139 (11.12)	2.7746 (11.89)	0.13
AVR	-2.5121 (9.44)	-0.3463 (6.89)	0.19
Fractal dimension	-0.3865 (2.92)	-0.3070 (1.03)	0.87
Arteriolar tortuosity $*10^5$	2.8953 (6.47)	-10.7576 (17.92)	0.04*
Venular tortuosity $*10^5$	5.3150 (11.71)	13.8332 (17.02)	0.77
Arteriolar branching angle	5.6844 (12.76)	-4.0759 (5.43)	0.10
Venular branching angle	-2.0723 (21.50)	0.7111 (14.80)	0.97
LDRa	2.69920 (70.035)	6.89026 (81.840)	0.44
LDRv	1.48275 (55.877)	-7.85749 (33.592)	0.33

All values are expressed as mean (SD), unless marked otherwise. BP: blood pressure, SBP: systolic blood pressure, DBP: diatolic bood pressure MAP: mean arterial pressure, CRAE-B: central retinal equivalent in zone B, CRAE: central retinal equivalent in zone C, CRVE(-B): central retinal equivalent in zone B or C, AVR(-B): arteriovenous ratio in zone B or C, LDRa: length diameter ratio arteriole, LDRv: length diameter ratio venule. [a]Values are expressed as median (interquartile range)

[b]Two participants had no Ambulatory BP measurements due to device malfunction.

Supplementary Figure 1

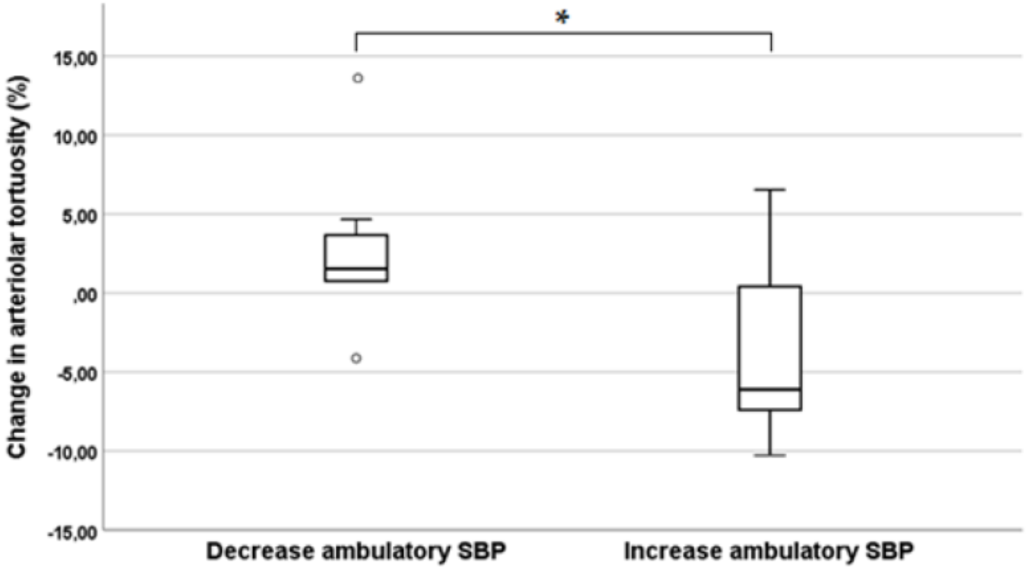

Supplement: Supplementary file 1 — Supplementary Information [file 41598_2020_79753_MOESM1_ESM.pdf]
